# The Cortical “Upper Motoneuron” in Health and Disease

**DOI:** 10.3390/brainsci11050619

**Published:** 2021-05-12

**Authors:** Roger N. Lemon

**Affiliations:** Department of Clinical and Movement Sciences, Queen Square Institute of Neurology, UCL, London WC1N 3BG, UK; r.lemon@ucl.ac.uk

**Keywords:** cortex, corticospinal, cortico-motoneuronal, movement

## Abstract

Upper motoneurons (UMNs) in motor areas of the cerebral cortex influence spinal and cranial motor mechanisms through the corticospinal tract (CST) and through projections to brainstem motor pathways. The primate corticospinal system has a diverse cortical origin and a wide spectrum of fibre diameters, including large diameter fibres which are unique to humans and other large primates. Direct cortico-motoneuronal (CM) projections from the motor cortex to arm and hand motoneurons are a late evolutionary feature only present in dexterous primates and best developed in humans. CM projections are derived from a more restricted cortical territory (‘new’ M1, area 3a) and arise not only from corticospinal neurons with large, fast axons but also from those with relatively slow-conducting axons. During movement, corticospinal neurons are organised and recruited quite differently from ‘lower’ motoneurons. Accumulating evidence strongly implicates the corticospinal system in the early stages of ALS, with particular involvement of CM projections to distal limb muscles, but also to other muscle groups influenced by the CM system. There are important species differences in the organisation and function of the corticospinal system, and appropriate animal models are needed to understand disorders involving the human corticospinal system.

## 1. A Critique of the Term ‘Upper Motoneuron’

Ever since Gowers (1886; see [1]), clinicians have used the term ‘upper motoneuron’ to distinguish clinical disorders affecting supraspinal structures directly influencing motor mechanisms from those affecting ‘lower’ cranial and spinal motoneurons. While it continues to be a really useful term in clinical description and diagnosis, the term is perhaps less useful in the context of modern neuroanatomy and neurophysiology, because UMNs in cortical lamina V have an extensive ‘connectome’ which includes not only their corticospinal (CS) projections, but also their extensive collateral projections to midbrain and brainstem motor structures, which in turn give rise to brainstem pathways influencing movement. These include the reticulospinal, vestibulospinal, and tectospinal tracts, which have a quite distinct spinal trajectory from the crossed corticospinal fibres. 

Further, the term UMN cannot be applied to all layer V corticospinal neurons because the corticospinal projection mediates functions other than somatic motor control, including autonomic control and control of peripheral somatosensory input [2]. Even for those UMN with motor functions, the organisation of UMN motor projections is strikingly different from that of lower motoneurons (LMNs; α-motoneurons in cranial and spinal motor nuclei), showing both convergence and divergence in relation to their target motoneuron pools. Whereas there is good understanding of the relatively fixed rules for recruitment of LMN during movement, the recruitment of UMN during natural movement is far more flexible and we only understand some of the rules. This should question the use of the term ‘upper motoneuron’ as a synonym for an M1 corticospinal cell, suggesting as it does a fixed and inevitable recruitment of that cell for a given movement, and further suggesting that recruitment of the UMN is a requirement for the subsequent discharge of the LMN.

In this review, I shall be concentrating on the primate corticospinal system and its CM subdivision [3]. It is important to state at the outset that these systems do not work alone during the execution of movement, but likely in combination with other brainstem and spinal motor systems [4].

## 2. Origins of the Corticospinal Tract Projection (CSP)

In addition to M1, a large number of frontal cortical areas contribute to the corticospinal tract, including premotor (PMv, PMd, SMA) and cingulate motor areas. Dum and Strick [5] determined the relative contributions to the CSP of these different projections, with the largest coming from M1 (50%) and large contributions from cingulate (21%) and supplementary motor areas (19%). Postcentrally, a large CSP arises from S1 and SII, with smaller numbers of fibres from areas 5 and 7. Unfortunately, no study has yet made a quantitative survey of the entire projection, so we do not know the exact proportions of all these different projections.

In relation to the overall cortical output, corticospinal projections, because of their direct actions on spinal motor mechanisms, represent a very influential system. On the other hand, compared with all the other corticofugal systems descending from the cortex, they are a numerically small fraction. Estimates based on the comparison of the cross-sectional area of the cerebral peduncle with that of the medullary pyramidal tract suggest that they number less than 10% of all corticofugal projections that arise from the cortex [6].

## 3. The Wide Spectrum of Primate Corticospinal Fibres

In most mammals, the CST is dominated by small corticospinal fibres, ranging in diameter from around 3 µm to as small as 0.5 µm [7,8]. Larger primates, including humans, possess, in addition, large-diameter fibres, up to around 12 µm in macaques and up to 22 µm in humans. The result is a remarkably wide spectrum of axon diameters in these primates, around one-hundred fold in the macaque [9]. Fine axons seem to arise from all cortical motor areas, while the largest fibres arise from area 4 [10], with a bias towards faster fibres from ‘new’ vs. ‘old’ M1 [11].

The really fast corticospinal fibres (conducting at >60 m/s) are very much in the minority, comprising a few per cent of the total, but with an importance out of proportion to their numbers. The larger corticospinal somas, which probably give rise to the thickest axons, tend to dominate pathological reports of human motor cortex, while studies using non-invasive stimulation have been mostly focused on short-latency MEPs, mediated by thick, fast-conducting axons. Interestingly, the fastest-conducting corticospinal neurons in the macaque are characterised by very brief action potentials [12], which may be related to the requirement for these neurons to fire at high instantaneous frequencies, for example, at movement onset. High velocities reduce conduction delays, which in a relatively large primate maybe important during transitions from movement to posture in skilled grasp [13].

We know very little about the overwhelming majority of corticospinal neurons with thin, slowly-conducting axons. They are largely missing from the primate neurophysiological literature [14], most likely because their action potentials are small and difficult to record stably with conventional recording techniques. Newer methods have revealed that it is possible to identify them [15], and therefore possible to begin to study their function.

We know that the larger CS fibres are particularly vulnerable to disease and to trauma, for example, during spinal cord injury [16,17]. There is also neuropathological evidence for selective impact of the neurodegenerative disease ALS on large corticofugal neurons in human motor cortex [18] (see below). In some autosomal recessive forms of the disease, there is evidence for preservation of a slowly-conducting CM system [19]. The contribution to pathology of the majority of fine axons in the CST demands further attention.

## 4. Corticospinal Projections and Terminations

The CSP from each cortical area terminates in a rather particular pattern within the spinal grey matter [20,21,22]. The diverse nature of the extensive cortical territory giving rise to the total CSP, combined with differences in fibre spectrum and in the pattern of spinal termination, all suggest that the CSP carries out multiple functions [2]. For example, there is a major contrast between the heavy CSP from the S1 hand representation (area 3b and 1) to the spinal dorsal horn [23,24] but completely avoiding the ventral horn, with the characteristic pattern of projections from the M1 hand area, which avoid the dorsal horn, but terminate heavily in lamina VII and amongst the motor nuclei of lamina IX [20,21].

While we still do not know what proportion of the CSP neurons make CM connections, we do know that terminations in lamina IX represent the second heaviest projection from M1 [21], and this suggests that the CM projection is a significant component of the M1 CSP, influencing motoneurons innervating flexors acting on the shoulder and elbow rostrally (C5–C7), along with flexors, extensors, abductors and adductors acting on the digits, hand and wrist caudally (C8–T1). Indeed, the proportion of all boutons, labelled in the motor nuclei of lamina IX of the lower cervical cord, after injection of tracer in the M1 arm/hand area has been calculated at around 18% [21]. This is likely to be an underestimate, since many boutons in adjacent lamina VII may also terminate on distal dendrites of motoneurons.

Morecraft et al. [21] found that almost all the arm/hand M1 projections terminate contralaterally. In contrast, projections from leg M1 give rise to bilateral projections [25].

## 5. Somatotopy in the Corticospinal Tract

As corticospinal fibres descend from the cortex in the corona radiata and internal capsule, fibres originating from the leg, arm, and face regions remain separated. However, by the time they reach the cerebral peduncle, there is more overlap and intermingling of fibres [26]. Since the time of Foerster [27], it had been postulated that there is somatotopy of fibres within the pyramidal and lateral corticospinal tract, with leg fibres lying laterally, and arm fibres medially; diagrams showing ‘lamination’ of the spinal portion of the CSP appear in several standard textbooks of anatomy and neurosurgery. The lamination idea is potentially of clinical importance because it has been used to try to explain some features of incomplete spinal injury in syndromes such as central cord syndrome, in which there is pronounced impairment of upper limb and particularly hand function but lower limb function is unimpaired [28]. This concept has been questioned on the basis of both anatomical and pathological findings. A recent systematic investigation by Morecraft et al. [29] provided unequivocal evidence against any lamination in the LCST, and instead suggested that incomplete spinal injury results in diffuse damage to the tract, which because of its greater influence over upper compared with lower limb function, often results in greater deficits of arm and hand movement.

Since it is clear that CST fibres heading to different spinal targets are intermingled within the spinal levels of the CST, these fibres cannot be using spatial cues to guide them to the correct target. Understanding the molecular identity of the different components of the CSP is a big challenge, but might explain its very targeted organisation and throw more light on its pathophysiology.

## 6. The Origin of the Cortico-Motoneuronal Projections

CM neurons are found in two main cortical areas: ‘new’ M1 and area 3a. Rathelot and Strick [30] coined the term ‘new’ M1 to define the caudal area of M1/area 4 which is restricted to the rostral bank of the central sulcus in the macaque (and, as area 4p, buried deep within the sulcus in humans [31]). Very few CM neurons were found in the rostral ‘old’ M1 region, on the convexity of the gyrus. This rostral region also gives rise to corticospinal projections that do not terminate directly on motoneurons. In addition, it projects to brainstem centres giving rise to other descending motor pathways, including the reticulospinal tract [7]. Stimulation of ‘new’ M1 can evoke fast, short-latency monosynaptic responses in macaque forelimb and hand motoneurons; no such responses were found from ‘old’ M1 [11]. Long-latency monosynaptic effects were evoked from both regions of M1, as were other more complex, oligosynaptic effects.

It is important to stress that CM effects do not arise only from large fast pyramidal neurons, as exemplified by the Betz cells. CM cells comprise a wide range of soma sizes [32], and CM effects from more slowly-conducting neurons have been reported [33].

The evidence for a CM projection in humans comes partly from neuroanatomical studies showing termination of corticospinal fibres amongst motoneuron pools [7,34,35], but mainly from analysis of motor evoked potentials (MEPs) generated by non-invasive electric (TES) or magnetic (TMS) stimulation of the motor cortex [36]. Some studies have used single motor unit recording to verify that these MEPs include discharge at delays that can be attributed to monosynaptic discharge of the motor unit after cortical stimulation [37,38]. TES and TMS of M1 have indicated that many upper and lower limb muscles receive CM projections in humans, with effects on upper limb muscles generally larger than those for the lower limb [39,40]. The cortical origin of CM projections in humans may be more extensive than in non-human primates. For example, there is some evidence for a CM projection from ventral premotor cortex in humans [41], which in macaques gives rise to corticospinal but not CM projections.

Importantly, TMS studies have revealed that there are pronounced differences in the extent of CM influence between muscle groups acting at different upper and lower limb joints. For example, effects on wrist extensors are stronger than those on wrist flexors, while foot dorsiflexors receive stronger effects than plantar flexors [37,38,42,43]. These differences may have important consequences in a variety of neurological syndromes, not least ALS (see below).

## 7. Organisation of the CM System as an Exemplar of the UMN

In this section, I want to consider some fundamental differences in the architecture of the CM cells and their projections, as exemplars of the UMN, when compared with that of their target α-motoneurons or LMN. To begin with, many CM cells *converge* onto the motoneurons innervating a particular muscle. Rathelot and Strick [30,32] reported the total numbers of CM cells labelled after a retrograde transneuronal virus was injected into a number of shoulder, elbow, hand, and digit muscles. Numbers varied from 158 to 814 CM neurons per muscle, and CM neurons innervating a given muscle were distributed over wide and overlapping regions of ‘new’ M1.

The CM system also shows *divergence:* individual CM cells, identified by spike-triggered averaging in the awake macaque, rarely show post-spike effects in a single muscle, but usually have a ‘muscle field’ comprising a number of different muscles [2]. The muscle field often includes a group of muscles which act together as ‘functional synergists’, i.e., they act together during complex behaviours such as reach and grasp, or during precision grip [44,45,46]. The same CM cell may exert inhibitory actions on muscle groups antagonist to their muscle field; these inhibitory actions are thought to be mediated by spinal inhibitory interneurons.

The LMN also has a divergent axon, such that it branches to innervate all the muscle fibres making up its motor unit. However, a given motor unit is normally innervated by only one motoneuron, so the unit does not receive convergent inputs. During muscle contraction, α-motoneurons are recruited according to a number of fixed rules, including the size principle, and discharge of an α-motoneuron, normally always leads to discharge and contraction of its innervated motor unit in a one-to-one manner. This is very different to the organisation of the input to the LMN from the CM and other pre-motoneuronal systems: typically many different convergent excitatory inputs must be active to bring the LMN to discharge, and each individual input produces a relatively small unitary EPSP. Therefore discharge of the LMN and the resulting movement requires synchronous input of many convergent excitatory inputs, probably including many of its CM neurons but also other supraspinal, propriospinal, and segmental inputs.

As pointed out above, there is no fixed pattern of recruitment between the UMN and the LMN. In an early study, Muir and Lemon [47] showed that macaque CM cells facilitating intrinsic hand muscles showed strong effects of task-specificity, with both CM cell and target muscle being active during one task (precision grip), but not during another (power grip). In the latter task, the CM cell was deactivated while the muscle was still active, and therefore the muscle must have been activated by inputs other than from the CM cell being recorded. Although most CM cells are ‘muscle-like’ in their pattern of activity, it is not a simple relationship [48,49]. Studies by Fetz [50,51] and by Schieber [52] have underlined the fact that the CM neuron and its target muscle can be readily dissociated by changing task conditions.

## 8. What Does the CM System Contribute to Skilled Movement?

Why should a direct projection from cortex to α-motoneuron have appeared late in evolution? A number of investigators have commented on the additional motor capacities that might be conferred by the CM system. One of the earliest ideas was that the CM system could act selectively on the motor apparatus of the upper limb to allow relatively independent digit movement [7,33,53]. Such movement is central to all skilled hand movements, including key capacities such as gesture and tool use. While tool use is not exclusive to advanced primates and humans, they do show a particularly large range of tool making and tool using behaviours, and CM projections are well developed in all Old World and New World monkeys that use tools [53,54]. Interestingly, CM cells have been shown to be strongly active during tool use by trained macaques [55]. CM cells are often active at the low force levels that characterise most skilled hand movements.

The CM system may also contribute to the production of particular patterns of muscular coordination that provide the biomechanical stability needed for force control at the most distal extreme of the skeleton, such as between the distal phalanges of the index finger and thumb. As pointed out above, CM cells generally show a ‘muscle-like’ pattern of activity i.e., resembles the timing and pattern of their target muscles, rather than being ‘extrinsic-like’ i.e., activity related to the direction of movement produced, independent of arm posture [49]. However, it is relatively uncommon for a CM cell to exhibit a pattern of activity similar to that of its target muscle when the muscle is employed as a simple agonist. Instead, individual CM cells can represent agonist, synergist, fixator, and antagonist functions of their target muscles [49]. Indeed, CM outputs organised along these lines show how this system operates to provide a great deal of functional flexibility in the manner of muscle recruitment, a flexibility that cannot be afforded by the relatively fixed synergies represented in spinal motor mechanisms [30].

## 9. The UMN and Amyotrophic Lateral Sclerosis

The involvement of the CST in ALS was known from the earliest descriptions of the disease [56]. The nature of that involvement is now increasingly being understood from both neurophysiological and neuropathological standpoints, which both confirm original suggestions that the CM component of the corticospinal system was implicated [57]. These aspects of ALS are covered in detail by clinical experts elsewhere in this volume, and in recent reviews [56,58,59].

I will just add a few additional points to the debate.

**Figure 1:**
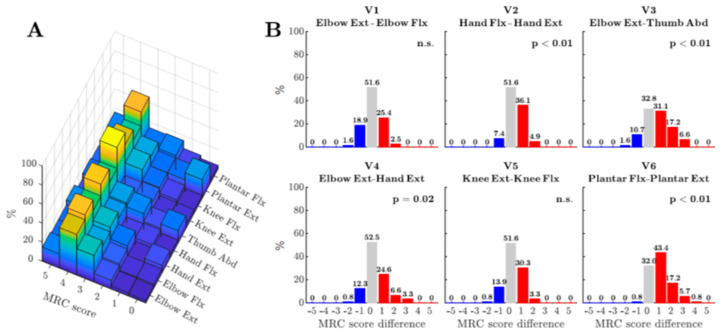
Pattern of weakness in upper and lower limb muscles in ALS patients. These data are from a prospective study of ALS in whom all 4 limbs were affected by the disease (*n* = 61 patients, *n* = 122 limbs). Colour scale is autoscaled to the maximum MRC score across all muscle groups. (**A**) Percentages of individual MRC scores for each muscle group. (**B**) Difference in MRC score between pairs of muscles for each muscle group. Bars indicate the percentage distribution of differences in MRC score. A positive difference (red bars) indicates that the muscle group receiving the less pronounced CM influence (first muscle group listed for each pair) was the stronger. Blue bars indicate a difference in the opposite direction. The *p*-value derived from the sign test of these differences is given at the top of each panel (from Ludolph et al. 2020 [68] Permission obtained).

1. A general concept on the function of the CM system is that it may subserve complex adaptive motor behaviours (including the examples of skilled tool use, but also vocalisation and advanced forms of locomotion), which obviously characterise much of the human motor repertoire [58,60]. It is striking that it is these same highly evolved behaviours that are affected in the early forms of the disease, as commented on over 130 years ago by Hughlings Jackson in his Croonian Lecture [61].
2. The split-hand syndrome, with dramatic differences in the strength of thenar vs. hypothenar intrinsic hand muscles is now recognised as a key feature in some forms of ALS [56,62]. This pattern of weakness appears to reflect loss of monosynaptic corticospinal inputs to the respective motoneuron pools that were first shown in the non-human primate, where stimulation of motor cortex was shown to evoke significantly larger CM EPSPs in thenar vs. hypothenar motoneurons [63]. For the intrinsic thumb muscles, it may be that the CM input represents such a large component of the excitatory input that when this input is compromised by ALS, the result is both a weakness and a poverty of movement. In the case of the split-hand syndrome, there is now good evidence that the pattern of weakness in ALS patients reflects changes in the corticospinal influence over these muscles, with clear changes in the short latency MEP [62]. This MEP is known to be dominated by direct CM action but may include other complex actions set up by the TMS pulse. Since investigation of the LMN in these patients suggests that peripheral changes do not make a significant contribution to the development of the split-hand phenomenon [64], this reinforces the importance of CM pathology in ALS.
  The accumulating evidence for cortical involvement in the early stages of ALS [18,58] is consistent with what we know about the degenerative processes affecting motor cortex pyramidal neurons with long corticofugal projections, including Betz cells. Recent work has particularly highlighted the loss of dendritic spines and altered synaptic inputs to the apical dendrites of Betz cells in post-mortem brains of patients with different forms of ALS, but not in other neurodegenerative diseases [65]. Similar changes have been seen in layer V pyramidal neurons in the cortex of TDP-43 mouse models of ALS [66,67]. It is further suggested that transmission of the pathogen pTDP-43 by the long axons of motor cortex corticofugal neurons results, in turn, in the degeneration of neurons in their subcortical targets. In particular, direct CM projections could provide a monosynaptic route for pTDP-43 transmission from motor cortex, resulting in degeneration of α-motoneurons in the brainstem and spinal cord [18].
3. Other recent studies have highlighted significant differences in the pattern of weakness in ALS patients which are consistent with the known connectivity of the CSP in humans and non-human primates [68,69]. An example is shown in Figure 1 from a recent study by Ludolph et al. [68], which included both retrospective and prospective studies of a large cohort of ALS patients whose muscle strength was assessed in different pairs of both upper and lower limb muscles, using the MRC scale. The study looked for any statistical sign of asymmetry, in each patient, between pairs of muscle groups, with one group known to receive strong CM connections and the other known to receive weaker connections. The pairs tested included wrist extensors vs. flexors, elbow flexors vs. extensors and, in the lower limb, knee flexors vs. extensors and plantar extensors vs. flexors. In the upper limb, strength was compared for a muscle group known to receive one of the strongest CM projections (thumb abductors) vs. one of the weakest (elbow extensors). The results showed a greater relative weakness for the muscle groups with normally receive strong CM projections, consistent with loss of CM influence during ALS [70]. These findings need following up, in a manner similar to that followed for the split-hand syndrome, to investigate the relative contribution of UMN and LMN to the weakness [59].
4. A neurodegenerative process selectively targeting, in its early phase, pyramidal neurons with long corticofugal axons, including CM projections, may result in an UMN syndrome that is different from that caused, for example, by acute stroke or spinal injury to the UMN and its axon. Kinnear Wilson [71] famously questioned the suggestion that ALS could be considered as a disease of the “pyramidal system” because although ALS patients exhibited “awkwardness of finger movements”, they only suffered from slight weakness and there was no spasticity. However, all the recent studies discussed above actually point to a consistent pattern of weakness in the hands and radial digits of ALS patients. Moreover, since selective damage to the CST is well known to result in poverty of hand and finger movement without spasticity [72,73,74], there is no need to invoke higher order motor deficits to explain the symptoms observed in early ALS, although such deficits may well play a part in later stages of the disease.
5. Progress in the understanding and treatment of ALS is dependent upon animal models. ALS appears to be a uniquely human disease and this has certainly made finding a good animal model difficult [18]. The involvement of the fast conducting CST and particularly of the CM system in ALS poses the question of whether a macaque model, whose motor system exhibits these same features, would allow additional insights to the progress already gained by the focus on rat and mouse studies [66,67,75], which do not share these features. Indeed, because in the rodent, corticospinal projections from sensorimotor cortex avoid the ventral horn and have limited direct effects on motor control [2,3], the layer V pyramidal neurons giving rise to these projections do not strictly qualify as ‘upper motor neurons’ in the sense that I have discussed in this short review. There are of course many features of motor cortex pyramidal neurons which are common to mouse, rat, macaque, and human [65,66,67,75], but we should also be aware that there are some striking differences, for example, in the shape and duration of their action potentials [12] and in some of the fast K+ ion channels involved in repolarisation [76]. These features would undoubtedly impact on the signals transmitted by the corticospinal tract and therefore on the different species-specific functions of this system.

## 10. Conclusions

The term UMN will continue to be useful in diagnosis of motor problems resulting from damage to the CNS. Its value will be enhanced if it is better understood in terms of the anatomical organisation and function of the different components of the motor system that ultimately influence the spinal and brainstem circuits that control movement. The UMNs share a flexible relationship with their target LMNs and reference to them as a sort of displaced α-motoneuron whose activity will lead in any simple or straightforward way to movement is erroneous. On the other hand, CM neurons serve as a useful example of system only onesynapse removed from the ‘final common path’, which has important implications both for function and for pathology. Learning more about the different sources of UMNs will undoubtedly teach us a lot more about the motor system in health and in disease.

## Data Availability

All data sets are presented within the papers cited.
